# Multidrug-resistant tuberculosis transmission among middle school students in Zhejiang Province, China

**DOI:** 10.1186/s40249-020-00670-x

**Published:** 2020-05-27

**Authors:** Yu Zhang, Lin Zhou, Zheng-Wei Liu, Cheng-Liang Chai, Xiao-Meng Wang, Jian-Min Jiang, Song-Hua Chen

**Affiliations:** 1 Zhejiang Provincial Center for Disease Prevention and Control, Hangzhou, Zhejiang China; 2 Key lab of Vaccine, Prevention and Control of Infectious Disease of Zhejiang Province, Hangzhou, Zhejiang China

**Keywords:** Multidrug-resistant tuberculosis, Transmission, School

## Abstract

**Background:**

Despite significant advancements in the treatment and diagnosis of tuberculosis (TB) over the past decade, drug-resistant TB remains an increasing threat to public health. TB outbreaks are most commonly reported in schools considering the delay in TB diagnosis, sustained contact, and overcrowding observed in schools. This report describes multidrug-resistant TB (MDR-TB) transmission in a school in Zhejiang Province. We aimed to raise awareness regarding MDR-TB transmission among students.

**Case presentation:**

The index patient was a 16-year-old girl in the second year of junior middle school in Zhejiang Province, China, who had been experiencing persistent cough and expectoration for 37 days since 1 March 2014. She tested positive for smear pulmonary and extrapulmonary TB on 8 April 2014 and was subsequently diagnosed with MDR-TB on 1 May 2014. However, the patient was resistant to isoniazid, rifampicin, ethambutol, and streptomycin. Thus, she was suspended from school for anti-TB treatment. All 54 students who were in close contact with the index patient in the same class were screened, and 5 tested positive on the tuberculin skin test. Their exposure time to the index patient was approximately 37 days. Three classmates were subsequently diagnosed with MDR-TB, with similar resistance profiles nearly two years later. Their average discovery delay was 55 days. These three classmates were also suspended from school for anti-TB treatment. During the treatment period, four students visited the local TB-designated hospital for further consultation every month and were followed up once a month by the local community health service center until they were completely cured.

**Conclusions:**

Discovery delay for an index patient played a primary role in MDR-TB transmission inside the school. To immediately detect TB, morning examinations in schools should be performed. TB trackers and case managers should work closely with public health workers and physicians in cases of TB outbreaks or transmissions involving students. Moreover, individuals who are in close contact with MDR-TB patients should undergo careful clinical follow-up for at least two years. Implementing a joint examination strategy to ensure early detection, diagnosis, and treatment of MDR-TB transmission is recommended.

## Background

Despite significant advancements in the treatment and diagnosis of tuberculosis (TB) over the past decade, drug-resistant TB remains an increasing threat to public health [1]. In 2018, approximately half a million new cases of rifampicin-resistant TB were reported, and 78% were diagnosed with multidrug-resistant TB (MDR-TB). The three countries with the largest share of the global burden of TB are India (27%), China (14%), and the Russian Federation (9%) [2, 3]. China is considered one of the countries with high MDR-TB burden. In 2018, according to China’s nationwide TB drug resistance survey, MDR-TB was reported in 7.1% of new cases and 21% of previously treated cases [3]. The available data indicate that individuals who are in close contact with MDR-TB patients who develop active TB most commonly have drug-resistant disease [3]. There are few studies assessing drug-resistant TB transmission among students, including studies conducted in California in 1981 and Mississippi in 1997 [4, 5]. However, these two studies did not assess MDR-TB transmission. Moreover, studies regarding drug-resistant TB transmission among students in China have not been conducted yet. To prevent MDR-TB transmission and provide a reference for MDR-TB control among students, we identified MDR-TB transmission during 2013–2017 among four students in one school in Zhejiang Province.

## Case presentation

### The index patient

The index patient was a 16-year-old girl in the second year of junior middle school in Zhejiang Province, China, who has experienced persistent cough and expectoration for 37 days since 1 March 2014. She tested positive for smear pulmonary TB and extrapulmonary TB (TB of the cervical lymph nodes) on 8 April 2014 (see Table 1). She was subsequently suspended from school and treated with chemotherapy comprising isoniazid (H), rifampicin (R), ethambutol (E), and pyrazinamide (Z) since 9 April 2014, with the dosages of these medications dependent on patient’s weight (see Table 2). During the treatment period (9 April 2014–8 November 2015), the index patient visited the local TB-designated hospital for further consultation every month and was followed up once a month by the local community health service center. However, the *Mycobacterium tuberculosis* isolate was resistant to H/R/E/S based on the results of the drug susceptibility test (DST) on 1 May 2014.

**Table 1:** Patients’ demographic and baseline characteristics

Code of Patient	Age^**a**^	Sex	Relationship	Complains of TB symptoms	Diagnostic time	Discovery delay^b^	Resistant pattern^c^	TST^d^	Treatment outcome
N1	74	Male	Neighbor	15 February 2011	21 April 2011	–	–	–	Failure
N2	61	Male	Neighbor	18 January 2012	18 February 2012	–	H/R/E/S	–	Failure
N3	64	Male	Neighbor	27 September 2013	9 October 2013	–	–	–	Failure
N4	59	Female	Neighbor	18 June 2015	15 July 2015	–	–	–	Failure
Index patient (Xu)	16	Female	–	1 March 2014	8 April 2014	37 days	H/R/E/S	–	Cure
P1 (Zhao)	15	Male	Classmates	15 January 2016	2 April 2016	77 days	H/R/E/S	Negative	Cure
P2 (Jiang)	16	Female	Classmates	12 March 2016	5 April 2016	23 days	H/R/E/S	Negative	Cure
P3 (Zhang)	17	Female	Classmates	1 December 2016	21 February 2017	81 days	H/R/E/S	Negative	Cure

^a^The record of the age of the patient was at the diagnostic time

^b^Discovery delay refers to the days between the time of diagnosis and symptoms

^c^Abbreviations: *H* isoniazid, *R* rifampicin, *E* ethambutol, *S* streptomycin, N1, N3, and N4 without DST results

^d^TST, tuberculin skin test; index patient, N1, N3, and N4 had no accepted TST test

-: not applicable

**Table 2:** Chemotherapy plan and dosage of multidrug-resistant tuberculosis in four students

Code of patient	Weight (kg)	Initial treatment		Multidrug-resistant treatment		Treatment end date	Course of treatment (days)	Complete the treatment and cure (Yes/No)
		Start date	Plan and dose	Start date	Plan and dose			
Index patient (Xu)	49	8 April 2014	H (7.5 mg/kg/day [8 h]), R (10 mg/kg/day [8 h]), Z (30 mg/kg/day [8 h]), E (25 mg/kg/day [8 h])	19 May 2014	Z (30 mg/kg/day [8 h]), Am (15 mg/kg/day [8 h]), Mfx (7.5 mg/kg/day [8 h]), PA (20 mg/kg/day [8 h]), Pto (15 mg/kg/day [8 h])	14 November 2016	951	Yes
				17 July 2014	Z (30 mg/kg/day [8 h]), Am (15 mg/kg/day [8 h]), PA (20 mg/kg/day [8 h]), Pto (15 mg/kg/day [8 h])			
				1 January 2015	Z (30 mg/kg/day [8 h]), PA (20 mg/kg/day [8 h]), Pto (15 mg/kg/day [8 h])			
P1 (Zhao)	46.5	2 April 2016	H (7.5 mg/kg/day [8 h]), R (10 mg/kg/day [8 h]), Z (30 mg/kg/day [8 h]), E (25 mg/kg/day [8 h])	22 June 2016	Z (30 mg/kg/day [8 h]), Am (15 mg/kg/day [8 h]), Mfx (7.5 mg/kg/day [8 h]), PA (20 mg/kg/day [8 h]), Pto (15 mg/kg/day [8 h]	25 July 2018	844	Yes
P2 (Jiang)	42	5 April 2016	H (7.5 mg/kg/day [8 h]), R (10 mg/kg/day [8 h]), Z (30 mg/kg/day [8 h]), E (25 mg/kg/day [8 h])	19 June 2016	Z (30 mg/kg/day [8 h]), Am (15 mg/kg/day [8 h]), Mfx (7.5 mg/kg/day [8 h]), PA (20 mg/kg/day [8 h]), Pto (15 mg/kg/day [8 h])	30 July 2018	846	Yes
P3 (Zhang)	35	21 February 2017	H (10 mg/kg/day [8 h]), R (15 mg/kg/day [8 h]), Z (30 mg/kg/day [8 h]), E (25 mg/kg/day [8 h])	20 May 2017	Z (30 mg/kg/day [8 h]), Am (15 mg/kg/day [8 h]), Mfx (7.5 mg/kg/day [8 h]), PA (20 mg/kg/day [8 h]), Pto (15 mg/kg/day [8 h])	7 November 2019	989	Yes

Abbreviations: *H* isoniazid, *R* rifampicin, *E* ethambutol, *S* streptomycin, *PA* pasiniazide, *Mfx* moxifloxacin, *Z* pyrazinamide, *Pto* propylthioisonicotinamide, *Am* amikacin

On 19 May 2014, the index patient was treated with regimens of moxifloxacin, propylthioisonicotinamide, amikacin, pyrazinamide, and pasiniazide (see Table 2). On 17 July 2014, as she experienced heel pain, the chemotherapy program was adjusted to regimens of propylthioisonicotinamide, amikacin, pyrazinamide, and pasiniazide. On 1 January 2015, the chemotherapy program was adjusted to regimens of propylthioisonicotinamide, pyrazinamide, and pasiniazide.

On 14 November 2016, she was subsequently cured according to the results of the laboratory smear and X-ray examination. During the treatment period, the sputum smear test results were negative in June, September, and October 2014.

Based on the interview conducted on the index patient, her classmates and teachers were her primary close contacts. Local health authorities conducted a contact investigation in April 2014. All potential contacts were screened by performing the tuberculin skin test (TST), and close contacts with positive TST results and with induration larger than 5 mm were diagnosed with MDR-TB [6]. The index patient’s two family members all had negative TST and X-ray examination results. There was no history of TB in her family. All 54 (49 classmates and five teachers) individuals with close contact with the index patient in the same class were screened during 12–15 April 2014, and five of these contacts had positive TST results. However, these five individuals refused to receive preventive anti-TB treatment. The five TST-positive contacts in our investigation had been consistently followed up, and based on the follow-up result, TB infection was no longer observed in the next two years in these five patients.

### Second-generation patients

In April 2016, two student patients (P1 and P2) tested positive for smear pulmonary TB in different high schools. P1 has persistently experienced the symptoms of cough and expectoration since 15 January 2016 and has left school because of winter vacation since 21 January 2016. P1 tested positive for smear pulmonary TB on 2 April 2016 and experienced hemoptysis, with multiple cavities in the lung parenchyma. His *M. tuberculosis* isolate was resistant to H/R/E/S based on the results of his DST on 22 June 2016. P2 has persistently experienced the symptoms of cough and expectoration since 12 March 2016, and she tested positive for smear pulmonary TB on 5 April 2016. She also experienced hemoptysis during that time. The *M. tuberculosis* isolate was resistant to H/R/E/S based on the results of her DST on 19 June 2016. They were suspended from school since 3 April 2016 and 6 April 2016, respectively, and were initially treated with chemotherapy comprising isoniazid (H), rifampicin (R), ethambutol (E), and pyrazinamide (Z), with the dosages of medications dependent on the patient’s weight. Subsequently, the chemotherapy program was adjusted to regimens of moxifloxacin, propylthioisonicotinamide, amikacin, pyrazinamide, and pasiniazide according to their DST results. All 56 (50 classmates and six teachers) individuals who were in close contact with P1 were screened through X-ray examination and TST during 6–8 April 2016. Moreover, all 58 individuals (52 classmates and six teachers) who were in close contact with P2 were screened during 7–11 April 2016. No other cases of TB or infection were observed.

On 2 February 2017, one student (P3) from another high school tested positive for smear pulmonary TB. P3 had persistently experienced symptoms of cough and expectoration since 1 December 2016. The *M. tuberculosis* isolate was resistant to H/R/E/S based on the results of her DST on 20 May 2017. She was also suspended from school since 22 February 2017 and was initially treated with chemotherapy comprising isoniazid (H), rifampicin (R), ethambutol (E), and pyrazinamide (Z), with the medication dosages dependent on the patient’s weight. Subsequently, the chemotherapy program was adjusted to regimens of moxifloxacin, propylthioisonicotinamide, amikacin, pyrazinamide, and pasiniazide according to the patient’s DST results. All 51 (46 classmates and five teachers) individuals with close contact with the patient were screened through X-ray examination and TST during 22–24 February 2017, and 14 of these 51 patients had positive TST results.

### Epidemiological investigation

Through a retrospective epidemiological investigation by local health authorities, there were no histories of TB in the patients’ families, although three student (P1, P2, and P3) patients clinically diagnosed with MDR-TB were from three different high schools in Lanxi City. However, they attended the same junior middle school and the same class as the index patient. Furthermore, they sat together in the classroom. The index patient sat in the middle of them, P1 was in the front of her, and P2, P3 were on her right and left side. Moreover, two of these patients (P2 and P3) lived with the index patient in the same dormitory (Table 1), and their exposure time to the index patient was approximately 37 days. Although they all had negative TST and negative X-ray examination results at that time, the drug resistance patterns of the four patients diagnosed with pulmonary TB were identical (resistant to H/R/E/S). Their average discovery delay was 55 days.

### Community investigation

**Fig. 1:**
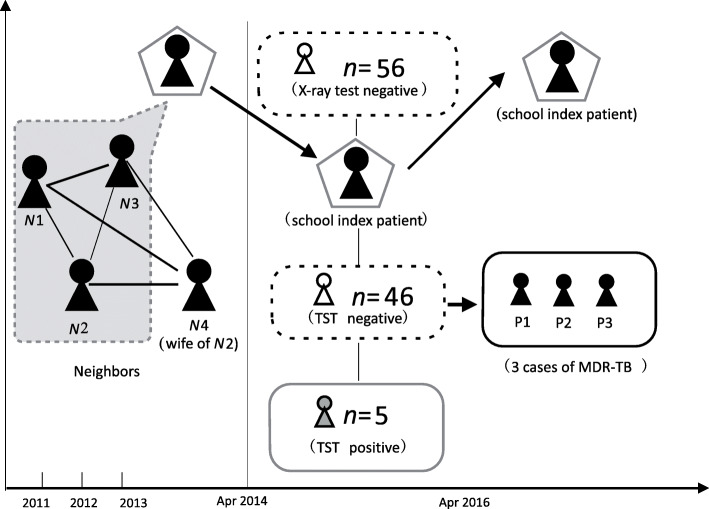
Hypothetical timeline of multidrug-resistant tuberculosis transmission in a school in Zhejiang, China, 2013–2017. The vertical dashed lines indicate the tuberculosis (TB) identified time of the index patient and the first secondary patient. The connection among cases was established through an epidemiological investigation. Neighbors with TB who are in close contact with the index patient are numbered N1 to N4. Secondary patients are numbered P1 to P3. Patients diagnosed with TB were colored black. Contacts with tuberculin skin test-positive results are colored gray.

After further retrospective epidemiological investigation, we learned that the index patient had evident risk factors for MDR-TB transmission. Epidemiological links were categorized into neighborhood environments. The neighbors (N1, N2, and N3) living around the index patient were all diagnosed with TB in 2011, 2012, and 2013, respectively (Fig. 1). N4, who was the wife of N2, was diagnosed with TB in July 2015. Four neighbors of the index patient experienced treatment failure due to irregular treatment, and three of them did not undergo further DST investigation. All neighbors were initially treated with regimens of isoniazid (H), rifampicin (R), ethambutol (E), and pyrazinamide (Z). Only N2 was diagnosed with MDR-TB and treated with regimens of moxifloxacin, propylthioisonicotinamide, ethambutol, pyrazinamide, and pasiniazide according to the patient’s DST results (resistant to H/R/E/S). However, he still experienced treatment failure due to the adverse effects of the medications and the heavy economic cost of treatment.

### Laboratory results

**Fig. 2:**
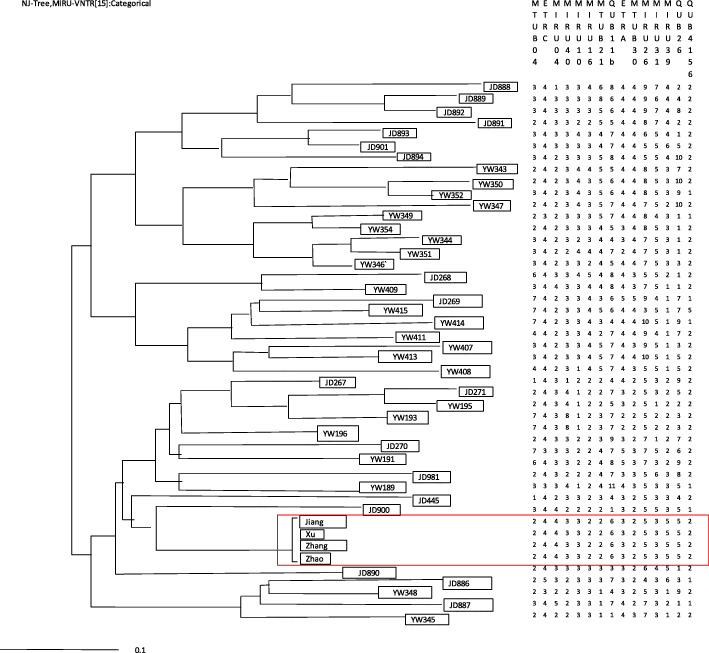
The results of MIRU-VNTR cluster analysis about 4 students with multidrug-resistant tuberculosis

Isolated strains from four patients (index patient, P1, P2, P3) were sent to Zhejiang Provincial Center for Disease Prevention and Control for repeat drug sensitivity tests and gene typing tests [7]. Variable-number tandem repeats of mycobacterial interspersed repetitive unit loci were used to genotype the strains. When 15 site combinations were used, the TB strains of four patients were clustered. The results showed that four patients had homologous transmission (see Fig. 2).

## Discussion and conclusions

TB outbreaks are most commonly reported in schools considering the delay in TB diagnosis, sustained contact, and overcrowding observed in schools [8, 9]. The results of this investigation indicate that discovery delay for index patients played a primary role in MDR-TB transmission inside schools. Students should be aware of the signs and symptoms of early TB, and physicians should consider the close contact of individuals to a TB-infected individual in the neighborhood as diagnostic evidence. Prompt TB diagnosis would have reduced the severity of illness and potentially prevented widespread school-based transmission. Furthermore, TST, a routine screening method, does not accurately identify the presence of latent TB infection. There is growing evidence that in low-incidence settings, currently available interferon-γ release assays (IGRAs) are less affected by bacillus Calmette-Guérin vaccination than the TST and that they are more specific and correlate better with exposure to an infected index patient [10, 11].

Some strains have developed tolerance to a variety of drugs and can be spread under specific conditions. The TB strains of the four student patients were clustered. It has been reported that TB strains with cluster distribution have strong virulence; hence, they can spread widely, resulting in a significant number of TB cases. Regarding MDR-TB transmission followed by a predictable pattern at school, students who were highly exposed to the index patient were at the highest risk of acquiring TB. Treatments were prescribed by infectious disease specialists at the local TB-designated hospital. Preplanned chemotherapy regimens based on personal DST results and adequate supplementary measures may have played important roles. These measures included incentives for both directly observed treatment-performing clinicians and patients and nutritional and emotional support. Patients were followed up daily until the end of follow-up, and drugs were administered under direct observation. Moreover, free psychological consultations and health education were also provided. We performed sputum smear, sputum culture, liver function, body weight, and blood routine examinations periodically to prevent adverse events. Additionally, 70 renminbi (the currency of China) nutritional subsidies and 70 renminbi traffic subsidies were provided to patients every month. In particular, offering free diagnoses and drugs may have ensured adherence. Thus, our supplementary measures might have produced a positive effect on compliance [12]. Hence, early MDR-TB detection and prompt initiation of treatment are important factors in determining the successful outcomes of TB treatment [13–15], subsequently preventing further MDR-TB transmission in schools.

This study has several limitations. The smear samples of patients (N1, N2, N3, and N4) in the investigation were not restored by the local hospital, and genotype investigation, which may further determine community MDR-TB transmission, was not conducted in these patients.

Several recommendations are made to reduce the risk of future MDR-TB outbreaks in schools. To immediately determine TB, morning examinations in schools should be performed. This was performed by checking whether each student had suspected symptoms of TB such as cough, expectoration, blood sputum, fever, and night sweats. IGRAs can be applied in the screening of individuals who have close contact with TB patients in schools, as appropriate [16–18]. Rapid diagnosis can be established using different tools such as the GeneXpert Mtb/RIF, which can be used to shorten the diagnosis delay of MDR-TB [19]. Individuals with close contact to MDR-TB patients should undergo careful clinical follow-up for a period of at least two years [18]. If active disease develops in MDR-TB contacts, prompt initiation of treatment with a regimen designed to treat MDR-TB is recommended. TB trackers and case managers should work closely with public health workers and physicians in cases of TB outbreaks or transmissions involving students to maintain the continuity of care among infected contacts.

## Data Availability

Data are available from the corresponding author upon request.

## Abbreviations

TB: Tuberculosis
MDR-TB: Multidrug-resistant tuberculosis
H: Isoniazid
R: Rifampicin
E: Ethambutol
S: Streptomycin
PA: Pasiniazide
Mfx: Moxifloxacin
Z: Pyrazinamide
Pto: Propylthioisonicotinamide
Am: Amikacin
DST: Drug susceptibility test
TST: Tuberculin skin test
LTBI: Latent tuberculosis infection
